# Investigation of an anatomically variant isolated bicuspid pulmonary valve: A case report: Erratum

**DOI:** 10.1097/MD.0000000000009683

**Published:** 2018-01-19

In the article, “Investigation of an anatomically variant isolated bicuspid pulmonary valve: A case report”,^[1]^ which appeared in Volume 96, Issue 52 of *Medicine*, the sentence on line 8 of page 3 should be written as “However, right ventricular hypertrophy in this case was not severe and decompensated cirrhosis was readily observed.”
